# The Cardiorespiratory Fitness of Children and the Anthropometric Determinants During Late Childhood Within South East Wales: Potential Implications for Future Health

**DOI:** 10.1177/2333794X241259908

**Published:** 2024-07-24

**Authors:** Stuart Jarvis, Helen Giles, Karl J. New

**Affiliations:** 1 University of South Wales, Pontypridd, Wales, United Kingdom

**Keywords:** cardiorespiratory fitness, anthropometry, children, physical fitness, population health

## Abstract

*Objective.* This study investigated cardiorespiratory fitness (CRF) levels and the relationship with field-based measures of anthropometry in children aged 10 to 11 years. *Methods*. A total of 288 boys and 257 girls participated in the study. CRF (20 m shuttle run) and several field-based measures of anthropometry were assessed. Multiple regression was utilized for all data analysis. *Results.* Boys performed significantly better than girls on the CRF test (*P* < .001) and achieved higher classifications of CRF based on centile norms compared to girls. All measures of anthropometry were significant predictors of CRF, (*P* < .001), and each measure was significant in predicting a negative trajectory of CRF performance when they increased in value (*P* < .05). *Conclusion*. The study findings add to the international reporting of CRF and the use of simple field-based measures of anthropometry alongside the use of BMI to predict CRF for health with Welsh school children (UK).

## Introduction

It has been suggested that having high cardiorespiratory fitness (CRF) in childhood and adolescents is associated with a range of health benefits such as better cardiovascular health, skeletal health, motor competence, cognitive ability, mental health, and self-esteem.^1^ Cardio-respiratory fitness refers to the capacity of the circulatory and respiratory systems to supply oxygen to skeletal muscle mitochondria for energy production needed during physical activity.^2^ Raghuveer et al^2^ highlighted that physical activity and CRF are often conflated but are indeed distinct but related concepts and Malina et al^3^ suggest that CRF may potentially increase or decrease, depending on one’s ability to be physically active. Unfortunately, levels of CRF in children and adolescents have declined over the past 6 decades.^4^ Tomkinson et al^4^ suggest that although the reasons for this are not well understood it may be attributed toward an increase in levels of obesity, an increase in the amount of sedentary time, a decrease in the levels of moderate to vigorous physical activity and a general reluctance of children and adolescents to engage in various forms of physical activity. It has been suggested that CRF levels are a stronger predictor of cardiorespiratory disease risk factors among youth than objectively measured physical activity levels.^5^ Raghuveer et al^2^ highlight that CRF is an objective measure of health that can be tracked over time whereas self-reported physical activity levels can be unreliable and only provide a snapshot of behavior. Further, longitudinal epidemiological studies have showed that physical fitness levels persist across the life course and that high CRF in childhood, adolescence, or early adulthood is prospectively associated with a healthier cardiovascular profile.^5^

Unfortunately, many countries still collect and report mainly on self-reported physical activity through national health surveillance systems.^1^ However Lang^6^ suggests that due to its importance, surveillance systems should be expanded to report on measures such as CRF. Lang et al^1,6^ further suggested that countries could further benefit from leveraging the measurement of physical fitness (CRF) to inform and track the effectiveness of policy and programming to improve the health of children and adolescents. In 2018, a global matrix of physical activity for children and youth across 49 countries highlighted that just over half (55%) of the included countries were unable to report on CRF due to the lack of available data.^7^ Therefore, there have been calls for the implementation of regular and consistent international and national fitness surveys to collecting data on CRF, similar to efforts conducted for physical activity which would in turn help better describe the global health status of children and adolescents.^1,6^

In addition to tracking levels of CRF it has been suggested that having better levels of CRF are correlated with various positive health indicators such as lower levels of obesity.^6,10^ Pereira et al^8^ suggested that children with adequate body mass index (BMI) and CRF levels were less likely to develop chronic diseases and more likely to display positive health outcomes. Therefore, considering the impact of BMI and CRF on children’s developmental health, Stoner et al^9^ suggested that it is vitality important to simultaneously measure these variables and potentially consider additional correlates that may influence these factors. In clinical practice and research relating to children and adolescence populations, BMI is still the most reported measure of weight status and obesity due to its simple invasive approach,^10^ but Lui et al^11^ advocates that considering the use of BMI alone is limiting due to its lack of reference to body fat. More recently, Domaradzka et al^10^ has suggested finding more than one simple to measure indicator which when combined with BMI will allow for a better estimation of health. Domardzka et al^10^ further outline that in recent years abdominal adiposis in children and adolescents has increased more than general adiposis which indicates that the frequency of obesity’s occurrence may be underestimated if it is based solely on BMI.

Unfortunately, the possibility to precisely assess body fat, especially central adiposity, requires specialist diagnostic equipment, hence in practice, to determine body fat distribution WC and WHtR and WHR are often used.^10^ Therefore, Aminianfar et al^12^ has suggested that studies which investigate population health, need to give ample consideration around anthropometric measurements, and include body height, body weight, body fat, body mass index, waist to hip ratio, waist to height ratio, and waist circumference in any screening as they are considered predictors of certain chronic diseases.

Considering the issues presented and their importance for promoting children’s health, the primary aim of this study was to investigate and report the levels of CRF in a cohort of Welsh primary school children. In addition, this study will also investigate a range of field-based measures of anthropometry and their relationship with CRF for consideration in any future health surveillance with children.

## Methods

### Participants

Participants were recruited to the study from primary schools located in a single unitary authority and based on their geographical and practical travel distance to the University test center. In total, 27 primary schools in South East Wales were invited to participate in the study, 18 of which accepted. Schools were briefed on the purpose of the study and issued with informed consent packs. Subsequent study inclusion criteria were based on an approach to parents and guardians of children enrolled in primary school years 5 and 6 (10-11 years) only. Only those participants returning signed parental consent, assent and fully completed and signed medical screening forms were considered for inclusion in the study. Conversely, the exclusion criteria included any participant with any pre-determined medical condition or physical impairment that could hinder them or the research investigation, participants who no longer wished to undergo evaluation prior to or on attending the test venue or subsequent withdrawal of consent/assent for their data set to be included in the final analysis.

A total of 640 completed consent packs were returned. Each school attended the test center at the university on separate dates and all data were collected in normal school operating hours during the summer school term. A total of 545 complete data sets were recorded for children aged 10 to 11 years old and compromised of 288 boys (*M* age = 10.94 years, SD = 0.56), and 257 girls (*M* age = 10.78 years, SD = 0.57). The participants were unintentionally exclusively Caucasian. All schools were from a single local authority in Wales and is classified as having 71.4% of its geographical area in the most deprived 50% of Wales.^13^

### Measures and Procedures

#### Cardiorespiratory fitness measurement

The participants CRF was assessed with one trial of the 20 m shuttle run test (20 m SRT), from the Assessing Levels of Physical Activity and Fitness (ALPHA) Health-Related Fitness Test Battery for Children and Adolescents Test Manual.^14^ The 20 m SRT has previously demonstrated moderate to high criterion related validity and high reliability of maximal oxygen uptake in 9 to 17-year-olds.^6^ The CRF assessment required the participants to run between 2 lines 20 m apart in time with an audio signal. The initial speed of the signal is 8.5 km/hour and is increased by 0.5 km/hour/minute The test finished when the participant failed to reach the end line concurrent with the audio signal on 2 consecutive occasions. Performance was recorded as the number of shuttles each participant completed. Each participant’s score for the 20 m SRT was then converted into a VO_2_ max value using the well-established prediction equation of Leger et al^15^ whereby the last stage number is used to predict maximal oxygen uptake (VO_2_ max, ml.kg^−1^.min^−1^) from the speed (X, km.hr^−1^) corresponding to that stage (speed = 8 + 0.5 × stage no.) and age (A, years): VO_2_ max (ml.kg^−1^.min^−1^) = 31.025 + 3.238X − 3.248A + 0.1536AX.

#### Anthropometric measurements

Anthropometric data collection included height, sitting height, waist circumference (WC), body weight and body fat (%). A portable floor standing Seca Stadiometer (Seca Birmingham, UK) measured height (cm) in bare feet to the nearest 0.1 cm. Sitting height was measured using the Seca Stadiometer to the nearest 0.1 cm. Body weight (kg) and body fat (%) was determined using the Tanita BC-418 MA device (Tanita, Middlesex, UK) to the nearest 0.1 kg. WC (cm) was determined by manual girth measurement on standing participants, using a Seca 201 tape measure (Seca Birmingham, UK) to the nearest 0.1 cm. BMI was calculated from body weight (kg) divided by height (m^2^) and the WHtR was calculated by dividing WC (cm) by height (cm).

#### Statistical analysis

Descriptive statistics and frequencies for the physical characteristics and performance measures were calculated. Prior to the applicable statistical analysis all data were checked to confirm the assumptions corresponding to linearity, homoscedasticity, multicollinearity, and normality. Regression analysis was used to identify if VO_2_ max values differed between genders. The VO_2_ max values obtained for each participant were classified into the 7-point adjective scale,^16^ with respect to centile norms specific to sex and age and the Mann-Whitney *U* test was used to determine any differences. Regression analysis was also used to determine correlations between the anthropometric measures and the results of the CRF test. Four models were created, in which each of the anthropometric measures (BMI, BF%, WC, and WHtR) was consecutively introduced as an independent variable. The models also considered the sex of the genders. Comparisons of the predictive value of anthropometric measures were made based on the coefficient of determination (*R*^2^). Finally, multiple regression analysis was run to provide an estimation of VO_2_ max variation from the anthropometric profiles of the participants. A significance level of *P* *<* .05 was applied to all analyses. All statistical analyses were performed using SPSS software, version 28.0 (SPSS Inc, Chicago, IL. USA).

#### Ethical approval and informed consent

Prior to the commencement of the study ethical approval was obtained from the human research ethics committee at the University of South Wales (Reference: 20014/HG, Faculty of Life Sciences). All parents or guardians of the participants, the participants and the participating schools were informed of the study’s objectives, and both written consent and assent forms were obtained prior to the start of the study.

## Results

### Descriptive Data

Descriptive study data for all anthropometric and CRF measures are reported in Table 1 for both boys and girls. The age distribution of boys and girls in the study was similar. Overall, there were minimal differences between gender on height. Across the anthropometrical measures selected girls presented higher profiles than boys for Body Weight (kg), BMI (kg/m^2^), Body Fat (%), and WC (cm) but showed no difference for WHtR. With regards to the measures of CRF the boys covered just over 10 more laps of the 20 m MSFT and demonstrated a slightly higher VO_2_ max value than the girls.

**Table 1.** Descriptive Data of Anthropometric and CRF Measures for Both Boys and Girls.

Variable (units)	Boys (n = 288)				Girls (n = 257)			
	*M*	SD	Min	Max	*M*	SD	Min	Max
Age	10.94	0.56	10.00	12.00	10.80	0.57	10.00	11.80
Height (cm)	143.47	7.15	125.2	171.2	143.01	7.78	124.7	167.0
Body Weight (kg)	38.86	9.41	23.7	84.0	39.32	8.45	22.3	65.3
BMI	18.70	3.26	13.38	32.97	19.09	3.13	13.47	31.55
BF (%)	21.24	6.16	12.7	46.1	25.51	5.20	16.8	48.1
WHtR	0.45	0.05	0.36	0.66	0.45	0.05	0.35	0.63
WC (cm)	65.48	8.68	51.2	105.0	64.8	7.77	50.1	89.2
MSFT (Laps)	41.26	20.12	3	99	30.65	15.48	3	81
VO_2_ max	49.11	5.02	37.9	62.90	46.78	4.02	40.00	58.30

Abbreviations: BMI, body mass index; BF%, body fat percentage; WHtR, waist to height ratio; WC, waist circumference; MSFT (Laps), laps of the 20 m multistage fitness test; VO_2_ max, maximum rate of oxygen.

### Classification of the Cardiorespiratory Fitness Between Genders

Overall, VO_2_ max values were significantly higher for the boys than girls *R*^2^ = 4.5%, *F* (1543) = 26.62, *P* < .001 with boys demonstrating a higher mean value of 1.62 mmol/L (95% CI, 7.56-13.65) than the girls. When VO_2_ max values were classified into the 7-point adjective scale (11) and with reference to the centile norms the median CRF percentile score remained significantly higher in the boys than in the girls, *U* = 26717, *Z* = −5.69, *P* = .001. On closer inspection (see Table 2), more boys tended to achieve a higher CRF classification based on the centile norms than the girls with just over 50% achieving a classification of good or above compared to only 28% of the girls. Of greater concern was that just over 50% of girls in the study were classified as achieving either fair or below levels of CRF compared to only 31% of boys.

**Table 2.** Classification of Cardiorespiratory Fitness (CRF) Regarding Gender and Centile Norms.

CRF classification	Total group (n = 545), N (%)	Boys (n = 288), N (%)	Girls (n = 257), N (%)
Very Poor (<P5)	25 (4.6)	13 (4.5)	12 (4.7)
Poor (P5-P19)	84 (15.4)	31 (10.8)	53 (20.6)
Fair (P20-P39)	110 (20.2)	46 (16.0)	64 (24.9)
Average (P40-P59)	108 (19.8)	51 (17.7)	57 (22.2)
Good (P60-P79)	110 (20.2)	66 (22.9)	44 (17.1)
Very Good (P80-P94)	82 (15.0)	60 (20.8)	22 (8.6)
Excellent (>P95)	26 (4.8)	21 (7.3)	5 (1.9)

### Anthropometric Measures to Predict Cardiorespiratory Fitness

It was demonstrated (Table 3) that all the measures of anthropometry were statistically significant with CRF. The coefficient of determination (*R*^2^) ranged between 60.1% and 69.1% with BMI% (*R*^2^ = 69.1%) followed by BF% (*R*^2^ = 65.3%), and WHtR (*R*^2^ = 61%) being the strongest measures to predict levels of CRF and slightly stronger than WC (*R*^2^ = 60.1%).

**Table 3.** Predictive Values of the CRF Test With Reference to Anthropometric Measurements.

Model	Independent factors	Statistics of regression models		
		*R* ^2^	*F*	*P*
1	Sex, BMI	69.1%	610.25	<.001
2	Sex, BF%	65.3%	513.08	<.001
3	Sex, WC	60.1%	410.23	<.001
4	Sex, WHtR	61.0%	424.17	<.001

Significance value set at (*P* < .05).

Abbreviations: *R*^2^, coefficient of determination; *F*, *P* test statistic and *P* value for significance of whole model; BMI, body mass index; BF%, body fat percentage; WC, waist circumference; WHtR, waist to height ratio.

In addition, to predicting which anthropometric measures were significantly correlated with CRF a multiple regression analysis was run to provide an estimation of VO_2_max variation from the anthropometric profiles of the school children. After independence of observations was observed, the subsequent analyses significantly predicted VO_2_max, *F* (4, 540) = 335.52, *P* < .05, adj. *R*^2^ = .711. All 4 of the anthropometric variables added significantly to the predictions, *P* < .05 (see Table 4). The results demonstrated that an increase in BMI of 1 kg/m^2^ it is predicted to be associated with a decrease in VO_2_ max of 0.65 ml/min/kg while an increase in WHtR of 1 unit of measurement also predicted a decrease in VO_2_ max of 0.56 ml/min/kg. The other measures of anthropometry predicted a decrease in VO_2_ max values, but these values were of smaller values.

**Table 4.** Multiple Regression to Predict VO_2_ max With Measures of Anthropometry.

		95% CI				
Variable	*B*	*LL*	*UL*	SE B	B	Sig
Constant	57.91	56.28	59.53	0.828		<.001
BMI	−0.65	−0.813	−0.495	0.081	−0.559	<.001
BF (%)	−0.24	−0.295	−0.186	0.028	−0.391	<.001
WC (cm)	0.06	0.004	0.114	0.028	0.129	.03
WHtR	−0.56	−1.238	0.114	0.344	−0.058	.03

Significance value set at (*P* < .05).

Abbreviations: *B*, unsaturated regression coefficient; CI, confidence interval; *LL*, lower level; *UL*, upper limit: SE B, standard error of the coefficient; B, standard coefficient.

## Discussion

The primary aim of this research was to add to the international reporting of CRF in Children. In the cohort of children who participated in this study within Wales (UK) it was shown that boys performed significantly better than girls on the test for CRF and achieved higher classification of CRF based on centile norms compared to girls. Our findings support similar research findings of Pereira et al^8^ and Ortega et al^17^ respectively in studies with boys and girls of similar age. In addition, to the reporting of CRF this study also considered the relationship of CRF values with measures of anthropometry for health surveillance in children and our findings demonstrated that all the measures of anthropometry (BMI, BF%, WHtR, and WC) were significant predictors of CRF. Each of the anthropometric measures were also significant in predicting a negative trajectory of CRF performance when each increased in value.

As previously highlighted, high CRF among children and adolescents is associated with a range of health benefits and CRF is a stronger predictor of cardiorespiratory disease factors among youth than objectively measured physical activity levels which track into adulthood.^1^ Globally, CRF has declined by 2.2% per decade from 1981 for all children and it has previously been suggested^18^ that we should use the interim international criterion- referenced standards of 35 and 42 mL/kg/min for girls and boys respectively to identify children and youth at risk of poor cardiorespiratory health and raise a clinical red flag.^14^ Although, more recently, Tomkinson et al^16^ among significant others proposed that we should be adopting a more specific age-related normative quintile-based framework to classify and track the CRF levels of children and adolescents.

Despite a plethora of data on children’s levels of CRF, Domaradzka et al^10^ recently suggested that there is still a greater need for additional studies of CRF in developed countries to establish a defined set of cut off points for CRF. There have also been calls by Lang et al^6^ to universally measure levels of CRF among children and adolescents for global health surveillance, monitoring and clinical screening and outline a greater need to refocus international efforts to identify the priorities that can help address major literature gaps and guide future physical fitness research and health surveillance. This has also been reinforced by Sandercock et al^19^ who also highlight that higher levels of CRF are associated with higher levels of physical activity and this has been shown to decline dramatically after age 11 therefore there is a greater need for frequent assessment, tracking and surveillance of CRF for health with children in late childhood (ie, 9-11 years) and into adolescence. Based on the frameworks proposed by Tomkinson et al,^15^ our study showed that 50% of girls and 31% of boys presented below average levels of CRF. It has been suggested by Stodden et al^20^ that possessing low levels of CRF in late childhood will negatively influence a child’s ability to persist in physical activity that require adequate levels of CRF. The authors of this study therefore believe they have provided some additional evidence on levels of children’s CRF albeit from a small sample of the childhood population in Wales (UK) to address some of these calls from the international community.

In addition to the monitoring of CRF in children and adolescence the measurement and surveillance of body composition is a public health norm. Internationally, the Organisation for Economic Co-Operation and Development (OECD) reported that the majority of the childhood population in the OECD area were overweight or obese and more specifically in the UK, it has been reported that childhood obesity levels at late childhood are still increasing and are of concern.^21^ It is also reported that childhood obesity prevalence has a strong negative correlation with levels of CRF in developed countries.^6,22^ The findings from our study also demonstrated support for this reported outcome and add to the call for it to be given greater consideration alongside key markers such as CRF for promoting children’s health.

The surveillance of body weight status at a population level is essential but there has been a growing call for research studies to adopt an additional measure which when combined with BMI will allow for a better monitoring of health risk. It has been suggested by Ross et al^23^ that WC and BMI should become a standard part of clinical encounters. In our study although marginal WC turned out to be the least sensitive predictor of CRF and instead the use of WHtR turned out to be a better predictor of CRF alongside the measurement of BMI. It has been suggested by Ashwell and Gibson^24^ that the use of WHtR can be a more effective indicator of early health risks related to central obesity and Alshamari et al^25^ also highlighted that WHtR is a much better predictor of cardiovascular disease than BMI and WC. Therefore, our findings although marginal add support to these previous authors suggestions.

### Strengths and Limitations

The strength of this study has specific applicability to Wales (UK). The Welsh Government^26^ has recently called for additional baseline data collection in the population to develop and further inform its Healthy Weight: Healthy Wales Strategy and impact on its nation’s health in the long term. At present in Wales there is very little measurement of both CRF and weight status in children of late childhood (ie, 9-11 years old). Wales currently only utilizes the Child Measurement Programme (CMP) in early childhood (ie, 4-5 years old). Therefore, the findings of this study will support the Welsh Governments calls for the extension of data within this specific population. The study also has limitations to note. First, in considering potential comparisons with other populations the data collected in this study is from a relatively small sample of children from one geographical area of Wales (UK), selected on proximity to the University test center. Therefore, caution must be exercised when interpretating the study findings as no a-priori power analysis calculation was conducted to justify the sample size used in this study. Second, due to its cross-sectional character, this study cannot determine the causality of the relationship between CRF and the measures of anthropometry and therefore this should be taken into consideration when interpreting the study findings. Finaly, maturational status was not considered as part of this study, but some participants may have entered this process, and this may have had an impact on the measurements undertaken.

## Conclusion

In conclusion, this study showed that boys performed boys significantly better than girls on the test for CRF and there were distinct differences between and across genders based on the CRF classifications. It also identified the use of simple field-based measures of anthropometry alongside the use of BMI to predict CRF for health. Therefore, the findings of this study will support the calls from the international community in the reporting of health and CRF in specific populations. In addition, it is also important to identify those children with poorer CRF and associated markers of weight status who may require additional support in the promotion of a positive health trajectory. Therefore, the use of simple, reliable, and valid field tests as used in this study could be performed by providers (ie, schoolteachers) in the future with little or no formal training in exercise physiology and in turn have the potential to reach large numbers of the population in the tracking and promotion of greater health.
